# Impaired tolerance to the autoantigen LL-37 in acute coronary syndrome

**DOI:** 10.3389/fimmu.2023.1113904

**Published:** 2023-03-27

**Authors:** Fernando Chernomordik, Bojan Cercek, Jianchang Zhou, Xiaoning Zhao, Nicole Wai Man Lio, Kuang-Yuh Chyu, Prediman K. Shah, Paul C. Dimayuga

**Affiliations:** Oppenheimer Atherosclerosis Research Center, Department of Cardiology, Smidt Heart Institute, Cedars-Sinai Medical Center, Los Angeles, CA, United States

**Keywords:** acute coronary syndrome, LL-37, T cells, immune checkpoint, self-antigen, platelets

## Abstract

**Background:**

LL-37 is the only member of the cathelicidin family of antimicrobial peptides in humans and is an autoantigen in several autoimmune diseases and in acute coronary syndrome (ACS). In this report, we profiled the specific T cell response to the autoimmune self-antigen LL-37 and investigated the factors modulating the response in peripheral blood mononuclear cells (PBMCs) of healthy subjects and ACS patients.

**Methods and results:**

The activation induced marker (AIM) assay demonstrated differential T cell profiles characterized by the persistence of CD134 and CD137, markers that impair tolerance and promote immune effector and memory response, in ACS compared to Controls. Specifically, CD8+CD69+CD137+ T cells were significantly increased by LL-37 stimulation in ACS PBMCs. T effector cell response to LL-37 were either HLA dependent or independent as determined by blocking with monoclonal antibody to either Class-I HLA or Class-II HLA. Blocking of immune checkpoints PD-1 and CTLA-4 demonstrated the control of self-reactive T cell response to LL-37 was modulated predominantly by CTLA-4. Platelets from healthy controls down-modulated CD8+CD69+CD137+ T cell response to LL-37 in autologous PBMCs. CD8+CD69+CD137+ T cell AIM profile negatively correlated with platelet count in ACS patients.

**Conclusions:**

Our report demonstrates that the immune response to the autoantigen LL-37 in ACS patients is characterized specifically by CD8+CD69+CD137+ T cell AIM profile with persistent T cell activation and the generation of immunologic memory. The results provide potentially novel insight into mechanistic pathways of antigen-specific immune signaling in ACS.

## Introduction

T Effector/Memory cells are present in specific stages of atherosclerotic cardiovascular disease (ASCVD) and its attendant risk of acute coronary syndrome (ACS). T Effector/Memory cell density is increased in ruptured plaques and decreased in plaques that have ruptured and resolved (1). Single cell RNA sequencing of peripheral blood mononuclear cells from acute myocardial infarction patients demonstrated inflammatory and effector characteristics of T cells (2). Peripheral T Effector/Memory cells correlate with atherosclerotic disease in both patients and animal models (3) suggesting involvement of antigen-specific immune memory in the disease etiology. In addition, clonality of plaque and thrombus T cells (4) suggest specificity to disease-relevant antigens. While generalized profiles of T cell responses in ACS patients have been informative (2, 5), specific activation markers in response to antigen stimulation (6–9) are now under investigation and may provide more clarity on their relevance to the clinical sequelae.

LL-37 is the only known human cathelicidin type antimicrobial peptide (6, 10). It is an autoantigen in psoriasis and other autoimmune diseases (11–15) that are known to increase the risk of cardiovascular events. It is a component of neutrophil extracellular traps (NETs) that are implicated in atherogenesis (16, 17). In addition, NET burden is associated with infarct size in ST segment elevation myocardial infarction (STEMI) (18) while LL-37 is associated with platelet activation and thrombosis (19). LL-37 is an immune modulator that has HLA-specific or adjuvant effects on the immune response (11, 20). Platelets are also immune modulators that regulate antigen-specific T cell responses (21). These factors contribute to the intrinsic T cell response to LL-37 but the context remains unclear.

LL-37 is an atherosclerosis self-antigen that provokes T Effector Memory response in ACS patients but not in stable ASCVD patients, suggesting that the self-reactive response may be more relevant in the acute stage (6, 10). However, the nature of the persistent presence of Memory T cells reactive to the self-antigen LL-37 in ACS patients compared to Controls and stable ASCVD patients remains unclear (6). LL-37 immune response is correlated with disease flare up and activity in autoimmune diseases (11–14). Thus, we evaluated T cell response to LL-37 in the context of healthy Controls and ACS patients using T cell immune activation, function, and memory markers. We evaluated the role of HLA dependent or independent responses. Immune checkpoints and the role of platelets as modulators of the self-reactive response were investigated. The results provide evidence supporting the impairment of immune tolerance to the self-antigen LL-37 in ACS.

## Methods

### Human PBMC

The protocol was approved by the Cedars-Sinai Institutional Review Board (IRB). Peripheral blood mononuclear cells (PBMCs) were isolated from blood collected from 21 patients with ACS within 72 hours of admission to the Cardiac Intensive Care Unit at Cedars-Sinai Medical Center. Patients were consented under the approved IRB protocol Pro00058160. Exclusions were inability to give informed consent, age less than 18 years old, active cancer treated with chemotherapy or radiation, patients taking immune-suppressive drugs, and pregnant women. PBMCs were isolated using Ficoll (GE Healthcare) density gradient centrifugation and cryo-preserved in commercially available cryogenic solution (Immunospot) in liquid nitrogen. Cryo-preserved PBMCs from self-reported healthy controls (N=16) were purchased from a commercial source (Immunospot).

### Activation induced marker assay of human PBMC

Cryo-preserved PBMCs were thawed, rinsed in anti-aggregation solution (Immunospot), and seeded in culture plates at a density of 3x10^6^ cells/ml of complete medium [RPMI 1640 medium (Invitrogen) supplemented with 10% heat-inactivated pooled human AB serum (Innovative Research) and 1X antibiotic/antimycotic (Gibco)]. After resting for 4 hours, cells were preincubated with 0.5 mg/ml anti-CD40 antibody for 15 minutes then stimulated with LL-37 peptide (20μg/ml) or CMV (pp65) Peptide Pool (StemCell Tech) (7, 22). Cells without treatment served as non-stimulated control. Cells were cultured in 37°C with 5% CO_2_ incubator and harvested 16 hours after stimulation, stained for viability (LIVE/DEAD Fixable Aqua Dead Stain Kit, Thermo Fisher), and subjected to cell surface staining for flow cytometry using the following antibodies: CD4, CD8, CD25, CD69, OX40 (CD134), 4-1 BB (CD137) and CD40L (CD154). Isotypes were used as staining control and CD14, CD16 and CD19 antibodies were used in dump gates to exclude B cells, dendritic cells, macrophages, granulocytes, eosinophils and neutrophils. Raw data are presented as percent AIM+ cells and are expressed as fold change (ratio between the antigen stimulated and the unstimulated condition) for each subject (7, 22, 23).

### CFSE proliferation assay

Aliquots of PBMCs (3x10^6^ cells/ml) were stained with 5µM CFSE in 37°C for 15 minutes, rinsed, and plated in complete medium. Cells were either stimulated with LL-37 peptide (20μg/ml) or vehicle (PBS). After 48 hours, complete medium was added at ^1^/_3_ volume and the cells were harvested 24 hours later for a total of 72 hours in culture. Collected cells were stained for flow cytometry using the following antibodies: CD3, CD4, CD8, CD45RO, CD62L, and CD197 (CCR7). Isotypes were used as staining control. Singlet, viable CFSE(+) cells were gated and CD4+ or CD8+ T Effector cells were further selected as CD45RO+CD62L(-)CD197(-). Results are expressed as Proliferation Index which is the ratio of LL-37 stimulated and No stimulation for each subject.

### CD107a cytolytic assay

PBMCs were seeded at a density of 3x10^6^ cells/ml in complete medium containing 2.5µg/ml mouse anti-human CD107a and stimulated with either 20μg/ml LL-37 or vehicle. After 1 hour, cells were treated with Monensin (1 x final concentration) and incubated for another 4 hours. Cells were collected and stained for CD8+ T Effector cells and analyzed by flow cytometry as described above. Data are reported as the ratio of LL-37 stimulation and no stimulation for each subject.

### Class-I or Class-II HLA blocking

PBMCs were seeded at a density of 3x10^6^ cells/ml in complete medium containing 20μg/ml LL-37. Each patient sample was plated in triplicates with one of the following conditions: no mAb treatment; 10µg/ml Class-I HLA mAb (mouse anti-human HLA A, B, C antibody; Biolegend); or 10µg/ml Class-II mAb (mouse anti-human HLA-DR; Biolegend). After 48 hours, medium was added at ^1^/_3_ volume and the cells were harvested 24 hours later for a total of 72 hours in culture. Collected cells were stained for flow cytometry using the following antibodies: CD3, CD4, CD8, CD45RA, CD45RO, CD62L, and CD197. Singlet, viable cells were gated and CD4+ or CD8+ cells further selected into T Effector cells as CD45RO+CD62L(-)CD197(-); T Effector Memory cells as CD45RO+CD62L(-)CD197(-)CD45RA(-); and TEMRA cells as CD45RO+CD62L(-)CD197(-)CD45RA+. The values are expressed as the ratio of HLA mAb blocking and no mAb treatment with values <1 considered HLA-dependent responses.

### PD-1 and CTLA-4 blocking

PBMCs were seeded in complete medium and stimulated with 20µg/ml LL-37 in triplicate for each patient with one of the following conditions: No treatment; 10µg/ml PD-1 mAb (BioXcel); or 10µg/ml CTLA-4 mAb (Ancell). Cells were collected after 16 hours and stained for flow cytometry using the AIM assay antibodies described above. For assessment of cytolytic activity, the procedure for CD107a assay was followed. The values are expressed as fold-change relative to no mAb blocking.

### Platelet co-culture with PBMC

Remnant blood samples from leukocyte reduction system (LRS) cones from platelet donors were acquired from the Cedars-Sinai Medical Center blood bank and subjected to PBMC isolation using a modified Ficoll technique as previously reported. Briefly, blood collected in the LRS cones were reconstituted with equal portion of PBS containing 2% pooled AB human serum and layered over SepMate Ficoll tubes (Stemcell Technologies). After centrifugation, the layer containing PBMCs and platelets was diluted into 50 ml PBS/2% pooled AB human serum and centrifuged at 350 x g for 10 minutes without brakes. The PBMC pellet was resuspended in culture medium and the supernatant containing the platelets was then centrifuged at 700 x g for 10 minutes without brakes to pellet the platelets. The platelet pellets were resuspended in 2 ml PBS/2% human serum and counted. Platelets were co-cultured with autologous PBMCs at a ratio of 25:1 (platelet:PBMC) with or without prior treatment with LL-37 for 30 minutes in a 37°C, 5% CO_2_ incubator. The cells were collected after 16 hours for AIM assay. Data are expressed as fold-change (ratio between the co-culture and the unstimulated condition) for each subject (7, 22, 23).

### CRP

CRP levels in patient plasma were determined using a commercially available ELISA kit (R&D Systems).

### Statistics

Data are expressed as mean ± SD. Data were tested for normal distribution and statistical significance was assessed using paired t test for normally distributed, paired data or Wilcoxon matched-pairs signed rank test for non-normally distributed, paired data. Student’s t test was used for non-paired, normally distributed data or Mann-Whitney U test for non-paired, non-normally distributed data. P < 0.05 was considered significant but trending data were also noted. For multiple cell population comparisons, significance was considered after Bonferroni correction. Pearson correlation coefficient was used to test the association between data sets with P<0.05 considered significant.

## Results

### Intrinsic T cell response to LL-37 in controls

**Figure 1:**
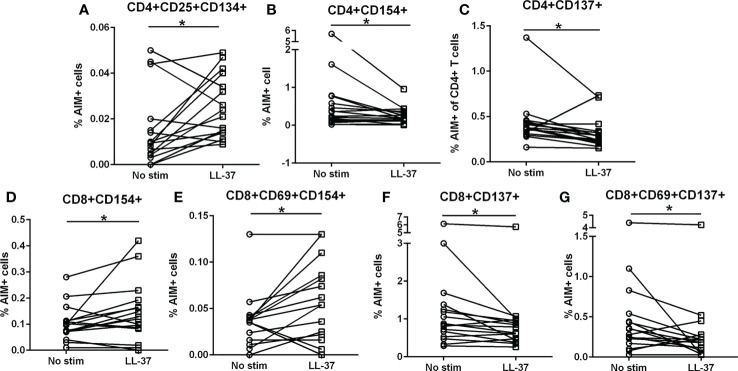
Intrinsic T cell response to LL-37 in Controls. PBMCs of Control subjects were stimulated with LL-37 for 16 hours. Activation induced marker (AIM) positive cells were compared between unstimulated and LL-37 stimulated CD4+ **(A–C)** and CD8+ **(D–G)** T cells. N=16; *P<0.05 paired t test or Wilcoxon matched-pairs signed rank test.

We have previously reported that LL-37 provoked a persistent T Effector/Memory response in PBMCs from ACS patients but not in stable CAD patients or healthy controls (6). To assess T cell activation profile in response to LL-37 as a self-antigen in PBMCs of Control subjects [N=16; Female=4 (25%)/Male=12 (75%); Age=60.0 ± 9.1], we tested for the presence of Activation Induced Marker [AIM](+) T cells (22–25). There was a significant increase in CD4+CD25+CD134+ T cells in response to LL-37 (**Figure 1A**) but significantly reduced CD4+CD154+ and CD4+CD137+ T cells (**Figures 1B, C** respectively). LL-37 also provoked increased CD8+CD154+ and CD8+CD69+CD154+ T cells (**Figures 1D, E**, respectively) but significantly reduced CD8+CD137+ and CD8+CD69+CD137+ T cells (**Figures 1F, G**, respectively) compared to no stimulation. No differences were noted in other activation markers. The results demonstrate the presence of T cells reactive to the self-antigen LL-37 in Control subjects characterized by increased CD4+CD25+CD134+ T cells, CD8+CD154+ T cells and CD8+CD69+CD154+ T cells. There is also a generalized reduction of CD137+ T cells.

### Intrinsic T cell response to LL-37 in ACS

**Figure 2:**
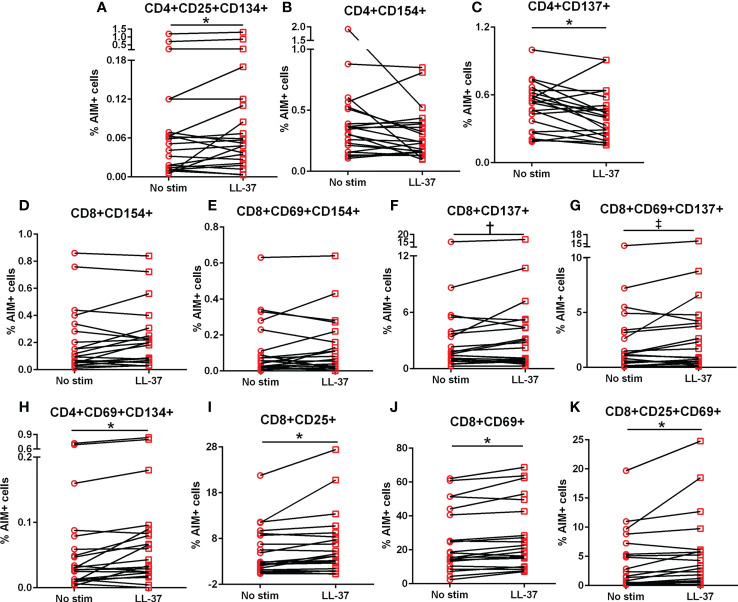
Intrinsic T cell response to LL-37 in ACS. PBMCs of ACS subjects were stimulated with LL-37 for 16 hours. Activation induced marker (AIM) positive CD4+ **(A–C)** and CD8+ **(D–K)** T cells were compared between unstimulated and LL-37 stimulated cells. N=21; *P<0.05; ^†^P=0.05; ^‡^P=0.06; paired t test or Wilcoxon matched-pairs signed rank test.

We then assessed the same activation markers in ACS PBMCs in response to LL-37. Patient characteristics are listed in **Table 1**. Similar to Controls, there was increased CD4+CD25+CD134+ T cells in response to LL-37 (**Figure 2A**) and trending lower CD4+CD154+ as well as significantly reduced CD4+CD137+ T cells (**Figures 2B, C**, respectively). Different from Controls, CD8+CD154+ and CD8+CD69+CD154+ T cells were unchanged (**Figures 2D, E**, respectively) but CD8+CD137+ and CD8+CD69+CD137+ T cells were trending higher (**Figures 2F, G**, respectively) after LL-37 stimulation. Additional activation markers were also increased in ACS that were not increased in Controls in response to LL-37: CD4+CD69+CD134+ T cells (**Figure 2H**), CD8+CD25+ T cells (**Figure 2I**), CD8+CD69+ T cells (**Figure 2J**), and CD8+CD25+CD69+ T cells (**Figure 2K**). The results demonstrate similarities and importantly differences in the T cell response to LL-37 in Control and ACS subjects.

**Table 1:** Patient characteristics (N=21).

Female/Male (%)	5 (24)/16 (76)
Age	61.9 ± 11.8
Diabetes (%)	4 (19)
Hypertension (%)	10 (48)
Dyslipidemia (%)	10 (48)
Smoker	
Current (%)	3 (14)
Past (%)	3 (14)
Statin use (%)	7 (33)
PCI (%)	21 (100)
1 vessel	12 (57)
2 vessels	4 (19)
3 vessels	5 (24)
WBC	10.9 ± 4.1
% Neutrophils	66.0 ± 16.7
% Lymphocytes	23.8 ± 14.2
Neutro/Lympho	5.2 ± 6.1
Hb	14.1 ± 2.5
Platelet	261.3 ± 61.7
Creatinine	1.0 ± 0.2
BUN	17.1 ± 5.2
Total Chol (mg/dL)	179.7 ± 55.5
LDL	113.7 ± 50.1
HDL	45.4 ± 10.7
Troponin first	7.9 ± 22.7
Troponin peak	88.8 ± 82.4
Troponin last	44.1 ± 41.0
LVEF % (echo)	48.0 ± 13.9
STEMI/NSTEMI	17/4
ADHF (%)	3 (14)
CVA	0
Repeat cath (%)	4 (19)
CRP (mg/L)*	4.4 ± 1.8

ADHF, acute decompensated heart failure; CVA, cerebrovascular accident; *CRP levels measured in 14 patients only due to sample loss.

### Standardized CD4+ T cell AIM in response to LL-37 stimulation

The data in **Figures 1** and **2** demonstrate differential T cell responses to LL-37 between Controls and ACS PBMCs. However, a standardized data analysis method is generally prescribed for AIM assay to account for inherent differences in baseline (unstimulated) PBMC profiles among subjects and thus data were expressed as fold-change relative to the unstimulated cells of each sample with a fixed activation threshold for functional relevance (23). This approach establishes a method to compare the magnitude of response between Controls and ACS. Although the activation threshold for non-self-peptide stimulation is widely accepted as functionally relevant at 2-fold change relative to unstimulated cells (22), a slightly lower threshold of 1.5 fold change is acknowledged for self-antigens (7).

**Figure 3:**
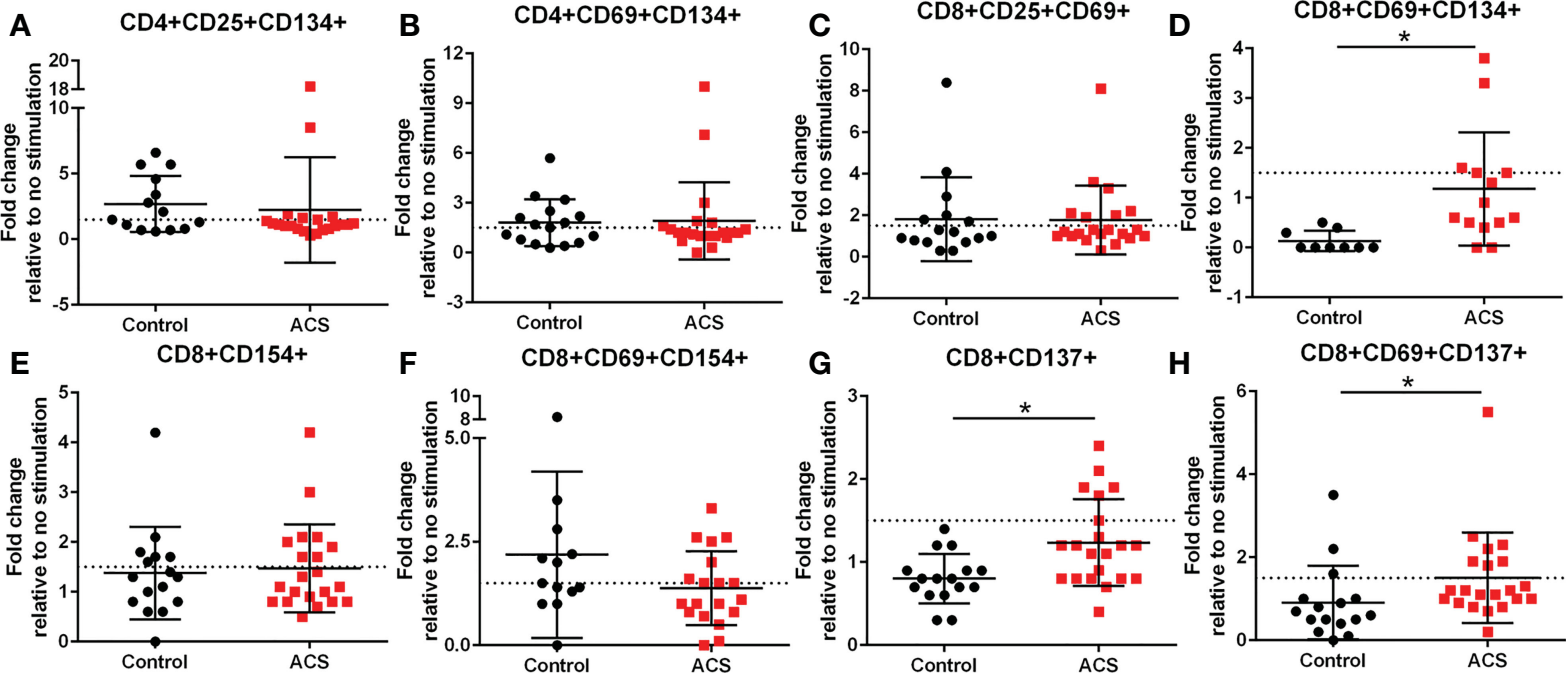
Activation Induced Marker (AIM) profile of T cells. PBMCs of Control and ACS subjects were stimulated with LL-37 for 16 hours. Data are presented as fold change in response to LL-37 stimulation relative to no stimulation for each subject. Dotted line represents activation threshold of 1.5-fold change in CD4+ **(A, B)** and CD8+ **(C–H)** T cells relative to no LL-37 stimulation. **(C)** *P=0.001; **(G)** *P=0.003; **(H)** *P=0.005; Student’s t test or Mann-Whitney U test.

The AIM profile demonstrated activation markers induced by LL-37 in PBMC T cells. CD25+CD134+ (**Figure 3A**) and CD69+CD134+ (**Figure 3B**) CD4+ T cells at or above the 1.5-fold threshold (dotted line) were observed in both Control and ACS subjects. The data presented as fold-change relative to no stimulation with a fixed threshold demonstrates a more stringent interpretation of the results. The results also suggest that the intrinsic response of CD4+ T cells to LL-37 in both Controls and ACS subjects is activation characterized mainly by increased CD134+ cells.

### Standardized CD8+ T cell AIM in response to LL-37 stimulation

LL-37 provoked AIM(+) CD8+ T cell response in both Controls and ACS PBMCs characterized by increased CD25+CD69+ cells (**Figure 3C**). However, CD8+CD69+CD134+ T cells (**Figure 3D**) were significantly reduced in Controls compared to ACS even as the mean for ACS did not reach the activation threshold. On the other hand, CD8+CD154+ T cells (**Figure 3E**) and CD8+CD69+CD154+ T cells (**Figure 3F**) were at or above activation threshold of 1.5-fold change in both Controls and ACS. CD8+CD137+ T cells (**Figure 3G**) were significantly increased in ACS compared to Controls even as it did not reach the activation threshold. Importantly, CD8+CD69+CD137+ T cells (**Figure 3H**) were significantly increased to activation threshold in ACS compared to Controls. Thus, the intrinsic response of CD8+ T cells to LL-37 in Controls is increased CD25+CD69+ cells yet characterized by reduced CD134+ and unresponsive CD137+ cells compared to ACS. The data suggest that although LL-37 reactive CD8+ T cells are present in Controls, the response is modulated by reduced CD134 and CD137 and this modulation is largely impaired in ACS. The results also demonstrate that CD8+CD69+CD137+ is the distinguishing T cell phenotype in response to LL-37 that fulfills the activation threshold and is differentially detected in ACS compared to Controls.

### LL-37 stimulation provokes T Effector cell proliferation

**Figure 4:**
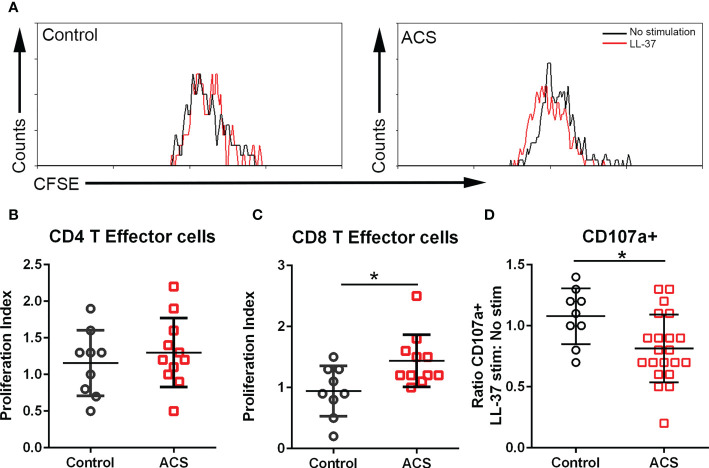
T cell proliferation and cytolytic activity. **(A)** Representative histogram of CFSE gated CD8+ T Effector cells to assess proliferation after LL-37 stimulation. **(B)** CD4+ T Effector cell proliferation index. **(C)** CD8+ T Effector cell proliferation index. **(D)** CD107a stain to determine cytolytic activity of CD8+ T Effector cells after 5-hour stimulation with LL-37. *P<0.05; Student’s t test.

The AIM assay data suggest that activation responses to LL-37 are mediated by CD134 and CD137. CD134 signaling mediates T cell responses that break tolerance (26) and CD137 enhances T Effector and Memory cell generation (27, 28). To determine if the T cell activation by LL-37 provoked Effector cell proliferation, PBMCs labeled with CFSE dye were stimulated for 72 hours and cell proliferation evaluated using CFSE signal decay (**Figure 4A**) expressed as the ratio between LL-37 stimulation and no stimulation (Proliferation Index). Cells were gated using T Effector markers (6). Stimulation of PBMCs with LL-37 resulted in marginally increased proliferation of CD4+ T Effector cells in both Controls and ACS (**Figure 4B**). On the other hand, CD8+ T Effector cells in Controls remained relatively unresponsive to LL-37 but proliferated in ACS (**Figure 4C**). The T Effector proliferation data are consistent with and confirm the results of the AIM assay, particularly the increased AIM(+) CD8+CD69+CD137+ T cells in ACS.

### Cytolytic activity of CD8+ T Effector cells in response to LL-37

CD8+ T Effector cytolytic function was evaluated using the CD107a assay. There was a significant reduction in CD107a+CD8+ T Effector cells after LL-37 stimulation of ACS PBMCs (**Figure 4D**). Thus, although LL-37 provoked T cell activation markers and increased T Effector cell proliferation, the cytolytic activity of these CD8+ T Effector cells was reduced.

### T cell AIM response to CMV peptides

**Figure 5:**
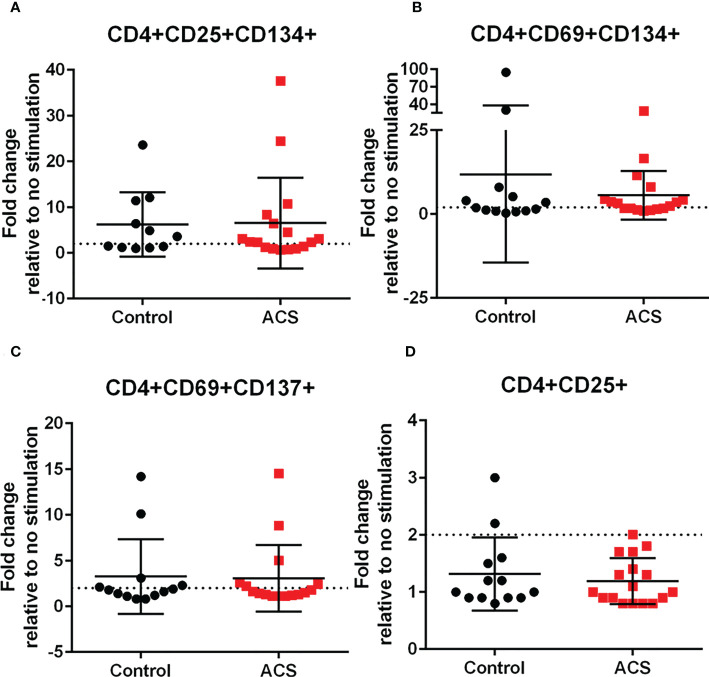
Activation Induced Marker (AIM) profile of CD4+ T cells in response to CMV pooled peptides. PBMCs of Control and ACS subjects were stimulated with CMV peptide pool for 16 hours. Dotted line represents activation threshold of 2-fold change relative to no CMV stimulation **(A–C)**. **D** was below threshold.

The results with LL-37 stimulation demonstrated intrinsic T cell response to a self-antigen that is implicated in innate inflammatory signaling and autoimmune conditions (11–14). The normal response in Control subjects that modulates this process is impaired in CD8+ T cells of ACS subjects. To assess if the responses observed is comparable to the responses to antigens from an infectious agent, PBMCs from the same subjects were stimulated with a CMV peptide pool. Pooled CMV peptides were used given the prevalence of CMV(+) adults in the general population (29). As such, immune reactivity and memory to CMV antigens in ACS remains the subject of investigations (30). Since the responses to CMV can be characterized as a recall response, it also served as validation of the AIM assay (22). Consistent with the reported activation threshold for infection and vaccine antigen stimulation, a 2-fold increase (22) (dotted line) in AIM(+) cells relative to non-stimulated cells was used. CMV peptide pool provoked various AIM(+) CD4+ cells in both Control and ACS subjects including CD25+CD134+ (**Figure 5A**), CD69+CD134+ (**Figure 5B**), and CD69+CD137+ (**Figure 5C**). CD4+CD25+ cells were below activation threshold with no difference in Controls and ACS (**Figure 5D**).

**Figure 6:**
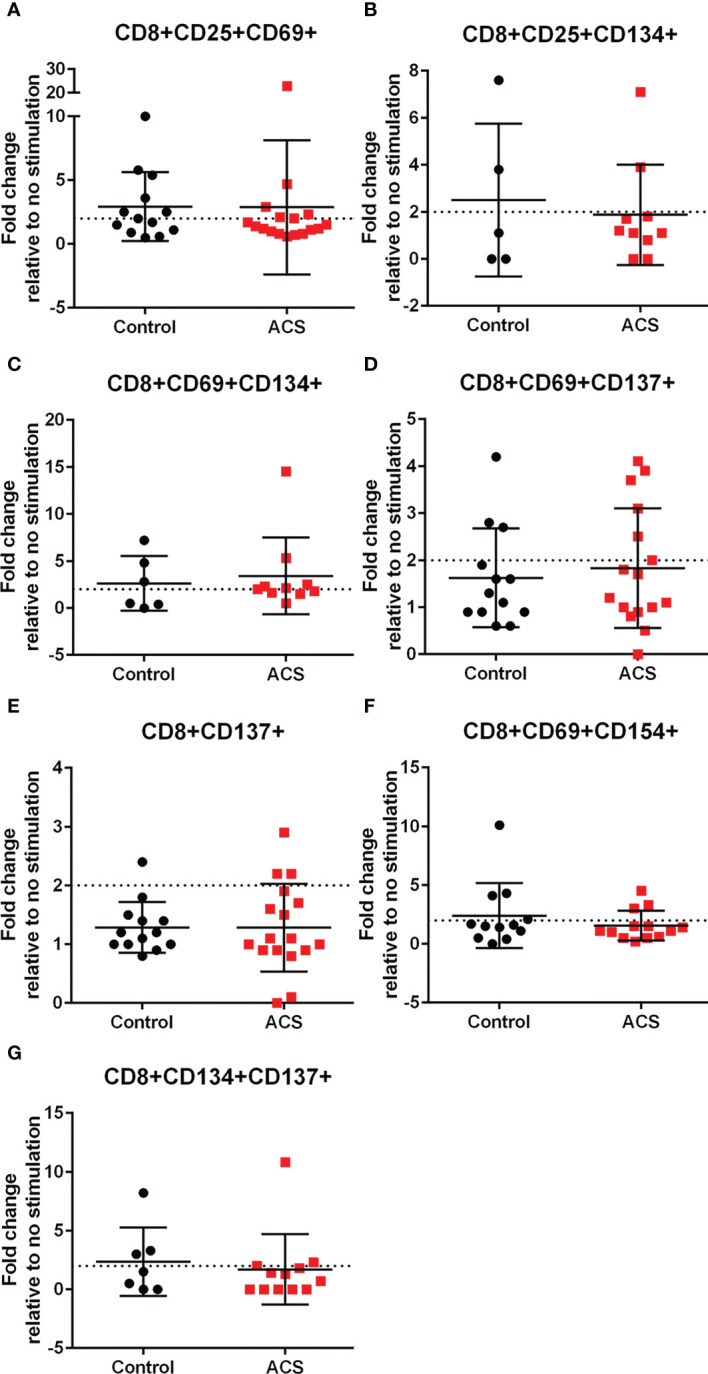
Activation Induced Marker (AIM) profile of CD8+ T cells in response to CMV pooled peptides. PBMCs of Control and ACS subjects were stimulated with CMV peptide pool for 16 hours. Dotted line represents activation threshold of 2-fold change relative to no CMV stimulation **(A–C, F, G)**. **D** and **E** were below threshold.

AIM(+) CD8+ T cells in both Control and ACS subjects were also observed, including CD25+CD69+ (**Figure 6A**), CD25+CD134+ (**Figure 6B**), CD69+CD134+ (**Figure 6C**), CD69+CD154+ (**Figure 6F**) and CD134+CD137+ (**Figure 6G**). CD8+CD69+CD137+ and CD8+CD137+ T cells remained below the activation threshold after CMV stimulation in both Controls and ACS (**Figures 6D, E**, respectively). None of the AIM profiles were different between Controls and ACS indicating that CMV provoked a differential T cell AIM profile compared to LL-37 stimulation.

### HLA dependent T effector/memory responses to LL-37 in ACS

**Figure 7:**
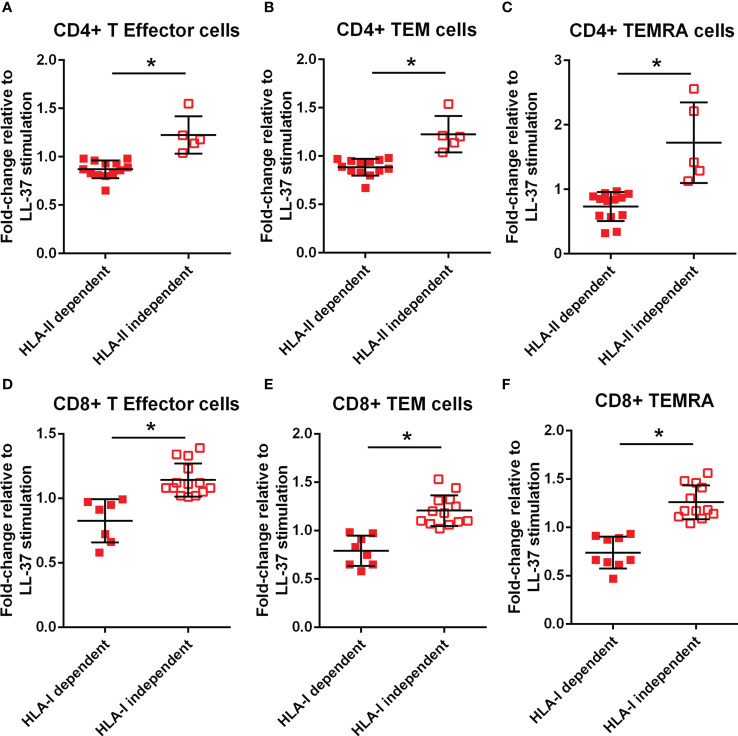
Monoclonal antibody blocking of HLA dependent T cell response. Effect of HLA-II monoclonal antibody (mAb) blocking on CD4+ T Effector **(A)**, T Effector Memory **(B)** and T Effector Memory RA+ **(C)** with LL-37 stimulation for 72 hours. Effect of HLA-I mAb blocking on CD8+ T Effector **(D)**, T Effector Memory **(E)** and T Effector Memory RA+ **(F)** with LL-37 stimulation for 72 hours. *P<0.05; Student’s t test.

To clarify if the signaling pathway involved in T cell responses to LL-37 is dependent on antigen presentation by HLA, monoclonal antibodies against Class-I or Class-II HLA were added to the culture medium at the same time the PBMCs were stimulated with LL-37. T Effector/Memory responses were expressed as the ratio of LL-37 stimulation with antibody blocking relative to LL-37 stimulation without antibody blocking and presented as fold change. Values lower than 1 would indicate blocking of HLA-mediated T Effector/Memory response. Not all samples had sufficient cells for the Class-II HLA blocking study. CD4+ T cells in majority of ACS samples tested were dependent on Class-II HLA mediated T Effector/Memory/RA+ responses to LL-37, with about 25% responding independent of Class-II HLA (**Figures 7A–C**). On the other hand, almost half of the samples had Class-I HLA dependent and just over half had Class-I HLA independent CD8+ T cell response (**Figures 7D–F**).

### PD-1 and CTLA-4 blocking results in perturbed T cell AIM profile

**Figure 8:**
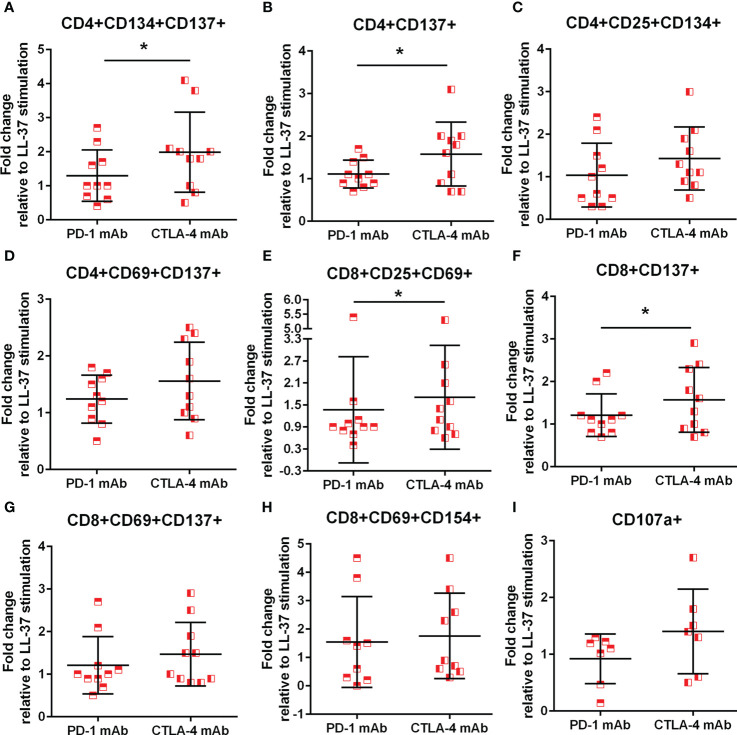
Monoclonal antibody blocking of PD-1 and CTLA-4. Effect of blocking immune checkpoint proteins PD-1 and CTLA-4 with monoclonal antibody (mAb) on CD4+ AIM(+) profile **(A–D)** and CD8+ AIM(+) profile **(E–H)**. *P<0.05; paired t test. Effect of mAb against PD-1 or CTLA-4 on cytolytic activity of CD8+ T Effector cells assessed by CD107a **(I)**.

Immune checkpoint proteins modulate T cell response to antigen stimulation and is particularly important in maintaining tolerance to self. The presence of LL-37 self-reactive T cells prompted the question whether such T cell response would be altered in the context of checkpoint inhibitor blocking antibodies during stimulation with LL-37. The results are presented as fold-change relative to LL-37 stimulation without blocking antibodies, such that no effect of blocking would be expressed as a value of 1. ACS patients have reduced PD-1 expression yet increased CTLA-4 expression that persisted compared to controls (31–33). Given the reported differential effect of inhibiting PD-1 compared to CTLA-4 (34), PD-1 blocking was compared to CTLA-4 blocking in the same patient samples. Using the AIM assay to assess checkpoint inhibitor blocking, there was a 2-fold increase in CD4+CD134+CD137+ T cells after CTLA-4 blocking in LL-37 stimulated ACS PBMCs that was significantly higher compared to PD-1 blocking (**Figure 8A**). CD4+CD137+ T cells were increased by 1.5-fold (**Figure 8B**) with similar trends for CD4+CD25+CD134+ and CD4+CD69+CD137+ T cells (**Figures 8C, D**, respectively) by CTLA-4 blocking. CD8+CD25+CD69+ and CD8+CD137+ T cells were also significantly increased by 1.5 fold in CTLA-4 blocking of ACS PBMCs stimulated with LL-37 (**Figures 8E, F**, respectively). CD8+CD69+CD137+ T cells were trending the same way but did not reach statistical significance (**Figure 8G**, P=0.06). CD8+CD69+CD154+ T cells were increased by PD-1 or CTLA-4 blocking by 1.5 and 1.8 fold, respectively (**Figure 8H**). The results suggest that although PD-1 antibody blocking increased T cell response in some ACS patient PBMCs, CTLA-4 blocking resulted in significantly perturbed T cell response to LL-37.

To assess functional effects of checkpoint inhibitor blocking during LL-37 stimulation, cytolytic activity by CD107a assay was also performed. There was a non-significant trend for a 1.5 fold increase in CD107a staining in CD8+ T Effector cells after CTLA-4 blocking compared to PD-1 blocking (**Figure 8I**; P=0.08), consistent with the preferential increase in AIM(+) cells after CTLA-4 blocking.

### Platelets modulate the T cell response to LL-37

**Figure 9:**
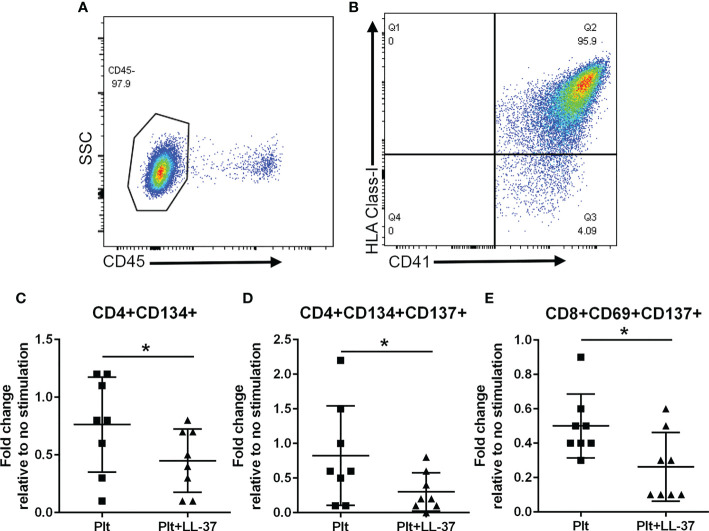
Platelets modulate the T cell response to LL-37. Platelets were isolated during PBMC collection from healthy platelet donors. Platelet enrichment was assessed by determining CD45(-) staining **(A)**. Over 95% of CD41+ platelets gated as CD45(-) stained positive for HLA Class-I [**(B)**, N=3]. Co-culture with platelets alone (Plt) or platelets with LL-37 stimulation (Plt+LL-37) demonstrated reduced AIM(+) T cell profile **(C–E)**. *P<0.05; N=8; paired t test or Wilcoxon matched-pairs signed rank test.

Platelet-rich arterial thrombus formation is the hallmark of ACS. Platelets are known to modulate T cells primarily by presenting antigens through Class-I MHC to CD8+ T cells (35) although other potential pathways that include modulating CD4+ T cells have also been reported (36). To investigate the potential role of platelets in modulating the intrinsic self-reactive T cell response to LL-37, we collected platelets during PBMC isolation from remnant samples of healthy platelet donors and assessed HLA Class-I expression. Platelet isolation yielded over 95% platelet enrichment (**Figure 9A**), majority of which expressed HLA Class-I (95.3 ± 0.8%; **Figure 9B**). Platelets were pre-treated with LL-37 for 30 minutes and co-cultured with autologous PBMCs for T cell AIM profile assessment. Data are expressed as fold-change relative to PBMC without stimulation. LL-37 treated platelets co-cultured with PBMCs significantly reduced CD4+CD134+ T cells, CD4+CD134+CD137+ T cells, and CD8+CD69+CD137+ T cells compared to platelets without LL-37 (**Figures 9C–E**, respectively). Thus, autologous platelets down-modulated the T cell response to LL-37 in healthy Controls. Importantly, this was observed in CD8+CD69+CD137+ T cells.

To evaluate the potential link between T cells and platelets in ACS patients, we assessed the relationship between CD8+CD69+CD137+ T cells, expressed as fold-change relative to no stimulation, and platelet counts. There was a significant negative correlation between CD8+CD69+CD137+ T cells and platelet count in ACS subjects (Pearson r = -0.438, P=0.047). This was not observed in CD4+CD134+ T cells and CD4+CD134+CD137+ T cells.

## Discussion

Our report demonstrates the novel finding that LL-37 provokes differential T cell response in Controls compared to ACS patients and preferentially provokes and significantly increases CD8+CD69+CD137+ T cells in ACS patients. In addition, we also report the following in ACS patients: 1) The AIM profile provides insight on signaling pathways that are involved in impaired tolerance response and in generating persistent immune memory to LL-37; 2) LL-37 provokes both HLA dependent and independent T cell responses; 3) Immune checkpoint protein CTLA-4 is an important regulator of T cell response to LL-37; and 4) The immune response to LL-37 as a self-antigen is distinct from the response to viral infection antigens from CMV.

Studies of T cell response in ACS have focused on increased CD4+CD28^null^ T cells (37) that have cytolytic activity (38) and natural CD4+ Tregs that are reduced and have impaired function in ACS patients (39). These are supported by the reports of clonal restriction of T cells in peripheral blood and in those acquired from coronary thrombi of ACS patients suggesting antigen-specific T cells (4). LDL (40) or its apolipoprotein component (7, 41, 42) and heat shock proteins (41, 43) are the focus of antigen specific response in atherosclerosis but investigations to characterize subpopulations of T cells in ACS are mainly without antigen specificity. However, autoimmune patients have impaired tolerance and are at higher risk for developing cardiovascular disease (44) suggesting potentially shared pathophysiology. Among the self-antigens of interest, we focused our work on LL-37, a T cell autoantigen in psoriasis and SLE (11–14), that plays a role in innate immune responses as damage-associated molecular pattern (DAMP) (45) as well as its participation in thrombus formation (19), both key elements of ACS. Increased LL-37 expression by intermediate monocytes in single-cell RNA sequencing assay of PBMCs was recently demonstrated in acute MI (2). We have reported the persistence of T Effector/Memory cells in PBMCs of ACS patients in response to LL-37 as compared to stable ASCVD and healthy controls (6), suggesting that increased adaptive immune response to LL-37 is present during the acute stage. Whether LL-37 and the immune responses provoked by it are functionally relevant in the pathophysiology of ACS remains to be determined.

### T cell activation induced markers in ACS

The AIM assay was developed to evaluate T cell responses to antigen challenge that remained below detection limits of the standard intracellular cytokine assays (22–24). The specificity of the assay has been validated by numerous reports characterizing T cell response to infections or vaccinations, but its use in evaluating self-antigens remains scarce (7). The results of the AIM assay were validated using pooled CMV peptides that provoked significant increase in most of the activation induced markers. This is in comparison to a select group of AIM(+) cells after LL-37 stimulation supporting specificity of the activation provoked by LL-37. The presence of CD4+CD25+CD134+ T cells and CD4+CD69+CD134+ T cells that were at or above activation threshold for both Controls and ACS in response to LL-37 is significant because CD134 ligation leads to the break of peripheral tolerance (26) that may be an important mechanism that drives LL-37 self-reactive immune response. Interestingly, there was reduced CD137 in AIM(+) CD4+ T cells of both Controls and ACS suggesting that the generation and expansion of Memory cells remains under control. This is supported by the marginal proliferation of CD4+ T Effector cells in response to LL-37 in both Controls and ACS. The context of how LL-37 provokes autoantigen CD4+ T cell response remains to be clarified. It is possible that the innate immune response to both trauma and infections that involve NETs generate the milieu that promotes self-reactive response to self-antigen DAMPs such as LL-37 (45).

The presence of CD25+CD69+ AIM(+) CD8+ T cells at or above activation threshold was also observed in both Controls and ACS that further supports the intrinsic T cell response to LL-37. In this context, the increase in CD134+ activated CD4+ T cells is important because another biological function of CD134 signaling is enhancing CD4+ T cell help towards CD8+ T cell response (46). The difference in CD8+CD69+CD134+ T cells between Controls and ACS can be characterized as more of a persistent presence in ACS as compared to the reduction in Controls, consistent with our prior report (6). The underlying cause of the persistent CD8+ T cell response to LL-37 is yet to be defined but T cell regulatory pathways are reduced in ACS (39, 47). This is evident in increased CD8+CD69+CD137+ T cells, as well as increased proliferative response to LL-37 by CD8+ T Effector cells in ACS. However, the complexity of the T cell response in ACS is highlighted by reduced CD8+ Effector cell cytolytic function compared to Controls. This aspect of the CD8+ T cell response is likely through CTLA-4 mediated mechanisms since blocking CTLA-4 resulted in increased cytolytic activity.

### HLA-dependent and independent T cell response

The context of HLA dependent compared to HLA independent immune response to self-antigens is underscored by its significance in tumor immune surveillance and evasion (48, 49). It is interesting that although the CD4+ T cell response to LL-37 in ACS is predominantly HLA Class-II, the CD8+ T cell response that can be attributed to HLA Class-I is only about 40% of the ACS patients in our study. The results highlight the potential role of LL-37 as an immune-modulator that is not limited to peptide antigens but also involving adjuvant functions that stimulate inflammatory signaling in APCs and other cell types (50). A non-HLA Class-I restricted activation pathway in response to LL-37 is through the specialized pro-resolving mediator (SPM) receptor FPR2, which is increased in ACS patient macrophages (2, 51) and mediates lipoxin A4 induced resolution of inflammatory signaling (52). LL-37 binding to FPR2 increases inflammatory signaling (53–55), and may alter T cell response through activation of other peripheral blood cells (2, 20, 56). It is thus important to emphasize that only a subgroup of ACS patients are HLA-restricted responders to LL-37.

### Immune checkpoint proteins and self-tolerance

We tested the role of immune checkpoints in regulating the T cell response to LL-37 in ACS. Both PD-1 and CTLA-4 blocking antibodies enhanced the AIM(+) T cell response to LL-37 with CTLA-4 antibody blocking provoking a stronger response. CD107a+CD8+ T cells were also increased by CTLA-4 antibody blocking indicating increased cytolytic activity. This is consistent with the reported immune related adverse events (irAE) that occur during immune checkpoint inhibitor (ICI) therapy. There was a higher incidence of irAE with Ipilimumab treatment which targets CTLA-4, compared to PD-1 or PDL-1 inhibitors (34). Our report demonstrates preferential modulation by CTLA-4 of the T cell response to LL-37 in ACS.

### LL-37 reactive T cell response is modulated by platelets

Platelet activation is a key step in athero-thrombosis in ACS (57) and platelets remain activated long after the acute event such that platelet function predicts recurrence of ACS (58). Platelet modulation of T cell response is attributed to their ability to present antigens in the context of Class-I MHC (35). Platelets are reported to down-modulate TCR mediated T cell activation through antigen presentation by denatured Class-I MHC or the lack of costimulatory signaling (21). Platelets reduced antigen specific CD8+ T cell response mediated by Class-I MHC in sepsis (59). Our study demonstrates the modulatory function of platelets in the response to LL-37 in healthy donors, which may be part of the mechanism that drives tolerance to LL-37 demonstrated in Control PBMC. We postulate that this mechanism is impaired in ACS patients, but we were unable to include platelet isolation during PBMC sample collection and thus could not test this. Nevertheless, the negative relationship between platelet count and LL-37 specific CD8+ T cell response in ACS suggests this may be an important mechanism that warrants further investigation.

## Conclusions

**Figure 10:**
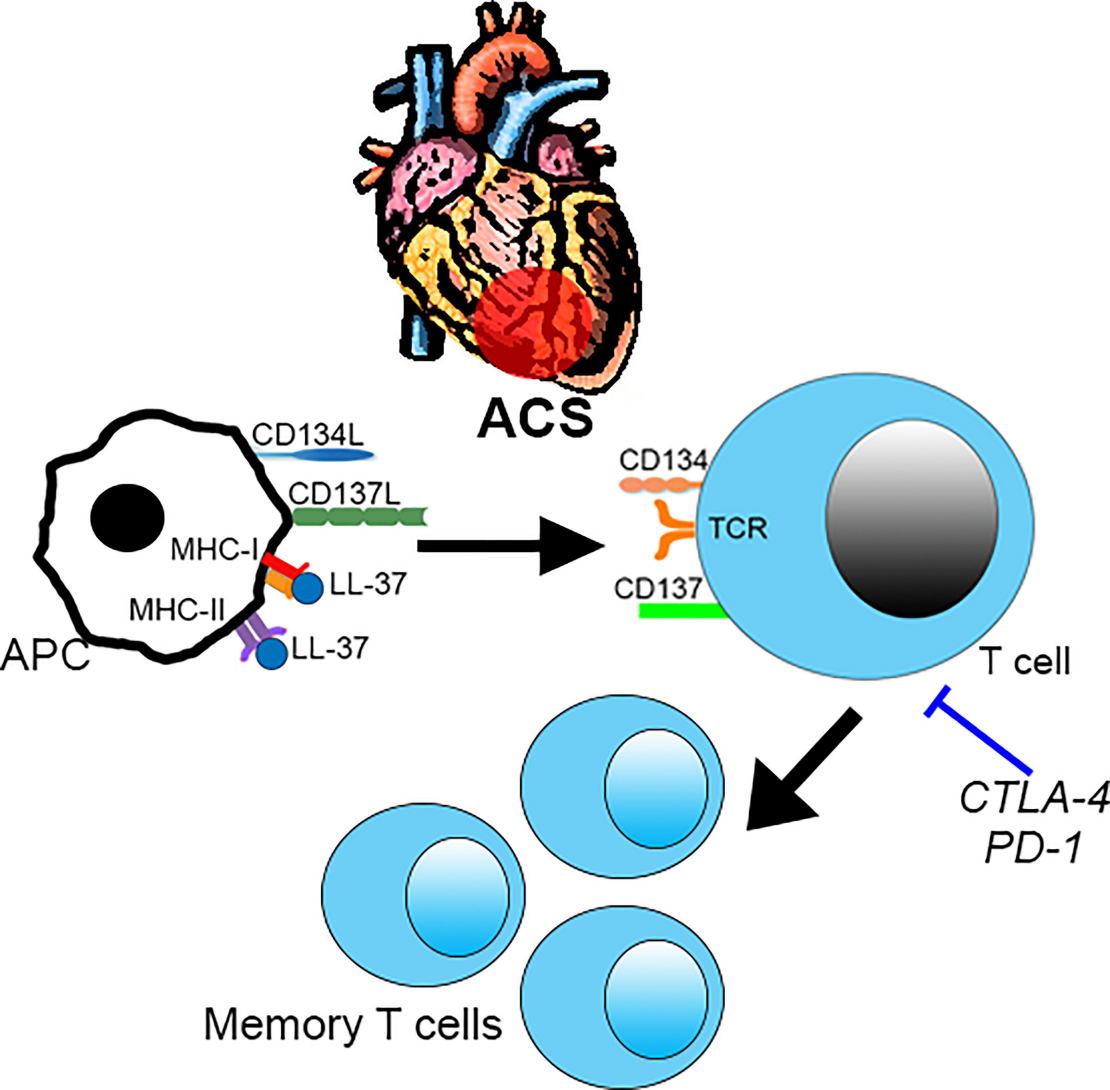
Intrinsic T cell response to LL-37 in ACS. T cell response to LL-37 in ACS patients is characterized by increased CD69 and co-stimulatory signaling through CD134 and CD137 that can potentially lead to breach of tolerance and persistence of self-reactive Effector/Memory cells. This is partially regulated by checkpoint proteins CTLA-4 and PD-1. .

The study extends our previous report on the potential role of immune-reactivity to LL-37 in ACS (6) by screening for not only activation induced markers but also potential pathways of immune signaling relevant to ACS. ACS patients were chosen for this study based on our previous report that patients with stable ASCVD did not have a robust immune response to LL-37 as compared to ACS patients (6). We have now confirmed the nature of the intrinsic T cell response in both Controls and ACS subjects. However, the small number of human subjects in the study is a limitation of our report. Another limitation is the cross-sectional design of the study, which cannot address its relevance to future events or if the immunologic memory to LL-37 persists over a longer period of time. Larger cohorts in longitudinal studies would be important to validate our findings. However, our study does provide the specific activation markers that can focus future studies and inform potential mechanistic investigations (**Figure 10**). We report that LL-37 provokes changes that impair tolerance and enhance immune memory generation in ACS characterized by CD8+CD69+CD137+ T cell activation profile. Platelets from healthy donors modulate the intrinsic response to LL-37. It remains to be determined what the implications are for platelets in ACS. Whether the presence of T cells responsive to LL-37 in ACS is detrimental also remains to be determined. In consideration of our current report, the role of immunologic memory to LL-37 as an autoantigen in ACS should be further investigated.

## Data availability statement

The original contributions presented in the study are included in the article/supplementary material. Further inquiries can be directed to the corresponding author.

## Ethics statement

The studies involving human participants were reviewed and approved by Cedars-Sinai Institutional Review Board. The patients/participants provided their written informed consent to participate in this study.

## Author contributions

Conception or design of the work: FC, BC, K-YC, PS, PD. Acquisition, analysis and interpretation of data: FC, JZ, XZ, NL, K-YC, PD. Drafting or revising work for critical intellectual content: FC, BC, K-YC, PS, PD. All authors contributed to the article and approved the submitted version.
